# Digital and Paper-Based Hospital Workflows and 60-Day Mortality in Acute Leukemia: Retrospective Natural Experiment

**DOI:** 10.2196/71306

**Published:** 2026-04-17

**Authors:** Christian Omar Ramos Peñafiel, Álvaro Cabrera García, Carolina Balderas Delgado, Rocío Luna Tentle, Andrea Milán Salvatierra, Nora Godínez Cubillo, Gustavo Acosta Altamirano, Rosa Sánchez Conejo, Adán Germán Gallardo Rodríguez

**Affiliations:** 1 Department of Hematology Hospital General de México Mexico City Mexico; 2 Department of Hematology Hospital Regional de Alta Especialidad de Ixtapaluca Ixtapaluca, State of Mexico Mexico; 3 Department of Hematology Hospital Juárez de México Mexico City Mexico; 4 Department of Management and Direction Hospital Regional de Alta Especialidad de Ixtapaluca Ixtapaluca, State of Mexico Mexico; 5 School of Sport Sciences Universidad Anáhuac México Huixquilucan, State of Mexico Mexico

**Keywords:** electronic medical records, traditional physical records, acute leukemia, 60-day mortality, Latin America

## Abstract

**Background:**

Electronic medical record (EMR) systems have been associated with better clinical workflows and fewer documentation errors. However, evidence regarding their effect on time-sensitive leukemia care in public hospitals in Latin America remains limited.

**Objective:**

This study aimed to compare 60-day mortality and urgent supportive-care processes between 2 tertiary public hospitals in Mexico with different documentation models (integrated EMR vs traditional physical records [TPR]), under the hypothesis that digital workflows may facilitate more timely treatment.

**Methods:**

We conducted a retrospective natural experiment including 274 patients with newly diagnosed acute leukemia treated between February 2023 and April 2025. Clinical characteristics, treatment intensity, complications during induction, antibiotic administration times, and survival outcomes were abstracted from finalized institutional records that are routinely reviewed at discharge by the institutional medical record committee as part of standard quality-assurance procedures. The primary outcome was 60-day mortality. Secondary outcomes included time to first antibiotic dose after recognition of febrile neutropenia, treatment-related complications, and the number of operational steps required for urgent care processes. A subgroup analysis of 70 patients with complete timing documentation was performed for antibiotic administration. Comparisons between hospitals were performed using univariate tests, Kaplan-Meier 60-day survival curves, and multivariable logistic regression.

**Results:**

Of the 274 included patients, 104 (38%) were treated at the hospital using an integrated EMR, and 170 (62%) at the hospital using TPR. Sixty-day mortality was lower in the EMR hospital (6/104, 5.8%) than in the TPR hospital (61/170, 35.9%; *P*<.001). In the subgroup with complete timing data, the mean time from febrile episode recognition to first antibiotic administration was shorter in the hospital using EMR than in the hospital using TPR (54, SD 18.4 minutes; *P*<.001). Although the EMR hospital used a higher proportion of high-intensity regimens, patients in that hospital had better 60-day outcomes. In multivariable analysis, hospital type remained independently associated with 60-day mortality (odds ratio 0.11, 95% CI 0.05-0.26; *P*<.001), whereas kidney injury and hepatotoxicity were associated with worse outcomes.

**Conclusions:**

In this natural experiment, the hospital using an integrated EMR had a lower 60-day mortality and shorter time to antibiotic administration than the hospital using TPR. These findings are hypothesis-generating and suggest that digital workflows may contribute to more timely urgent supportive care, but they should be interpreted with caution, given the retrospective 2-center design and the potential for residual confounding.

## Introduction

Electronic medical records (EMRs) are digital patient records that integrate clinical history, laboratory results, diagnoses, treatments, and medication orders. Compared with traditional physical records (TPR), EMRs may improve traceability, facilitate communication among physicians, nurses, pharmacists, and ancillary services, and reduce delays in the execution of urgent clinical decisions [1,2].

Over the last 2 decades, digitalization has transformed hospital care in many high-income settings. In the United States, widespread adoption accelerated after the Health Information Technology for Economic and Clinical Health (HITECH) Act, and similar transitions have occurred in several European countries [3]. In oncology, digital tools have been associated with better coordination of care, improved remote monitoring, fewer unplanned admissions, and more timely responses to complications [4].

Despite these advances, Latin America continues to face major barriers in cancer care, including fragmented health systems, limited access to innovative therapies, administrative delays, and marked social inequities [5,6]. These limitations are especially relevant in hematologic malignancies, where delays in diagnosis, treatment initiation, and supportive care can have an immediate impact on survival [7].

In acute leukemias, early mortality remains a major challenge in the region. Patients with acute lymphoblastic leukemia (ALL) and acute myeloid leukemia (AML) are highly susceptible to infectious and hemorrhagic complications during the first weeks of treatment [8-12]. In Mexico, delays from symptom onset to treatment initiation have been associated with poor outcomes, and infectious complications are a leading cause of early death [13]. In acute promyelocytic leukemia, hemorrhage remains an additional major contributor to early mortality [14].

A substantial proportion of these delays occur not only at the diagnostic stage but also during hospital care, particularly when urgent interventions, such as antibiotic administration or blood component delivery, depend on multiple manual steps [15]. In many public referral hospitals in Mexico, these processes still rely on paper charts, handwritten requests, and fragmented communication pathways.

Therefore, we treated the contrast between these 2 hospitals—Hospital Regional de Alta Especialidad de Ixtapaluca and Hospital Juárez de México—as a natural experiment. Our aim was to compare 60-day mortality and time-sensitive supportive-care processes between a fully digital public hospital and a hospital using TPR, while also exploring clinical predictors of mortality. We hypothesized that EMR-supported workflows would be associated with faster delivery of urgent supportive care and lower 60-day mortality in patients with newly diagnosed acute leukemia.

## Methods

### Setting

We conducted a retrospective natural experiment, a type of observational study, of patients with newly diagnosed acute leukemia treated between February 2023 and April 2025 at 2 tertiary public hospitals in Mexico. The comparison included Hospital Juárez de México, which uses a TPR model with paper-based requests and manual delivery processes, and Hospital Regional de Alta Especialidad de Ixtapaluca, which operates with a fully digital infrastructure, including EMR-based prescribing, real-time medication management, and electronic request processing. Acute leukemia diagnosis was established using the standard institutional diagnostic workup, including morphological evaluation of peripheral blood and/or bone marrow together with immunophenotyping by flow cytometry. The French-American-British classification, when documented in the record, was used only as a descriptive morphological framework.

### Participants

Eligible patients were consecutive adolescents and adults with a confirmed diagnosis of acute leukemia who received inpatient induction or initial inpatient antileukemic treatment during the study period. Patients were included regardless of leukemia subtype if the diagnosis and inpatient treatment course were documented in the institutional record. Patients without vital-status information through day 60 or with missing primary outcome data were excluded from the primary analysis.

### Data Collection: Data Sources and Variables

Data were retrospectively abstracted from finalized institutional records at each center using a predefined study dataset. Data abstraction was performed by trained investigators involved in the study using standardized extraction forms developed for the project.

At both hospitals, the medical record of each hospitalized patient is routinely reviewed at discharge by the institutional medical record committee as part of standard administrative and quality-assurance procedures; thus, the study used records that had already undergone routine institutional review. In the TPR hospital, study variables were obtained from paper charts, handwritten medical and nursing notes, medication sheets, and discharge documentation. In the EMR hospital, the same variables were obtained from the digital platform used for clinical documentation, medication tracking, and time-stamped orders. Collected variables included age, sex, leukemia subtype, baseline white blood cell count, prior infection, overweight status (BMI **≥**25 kg/m^2^), treatment intensity, treatment-related complications, organ toxicities, and vital status at 60 days. No formal duplicate abstraction or interrater reliability exercise was performed for the research dataset, which should be considered when interpreting the results.

### Outcomes and Treatment Classification

**Figure 1:**
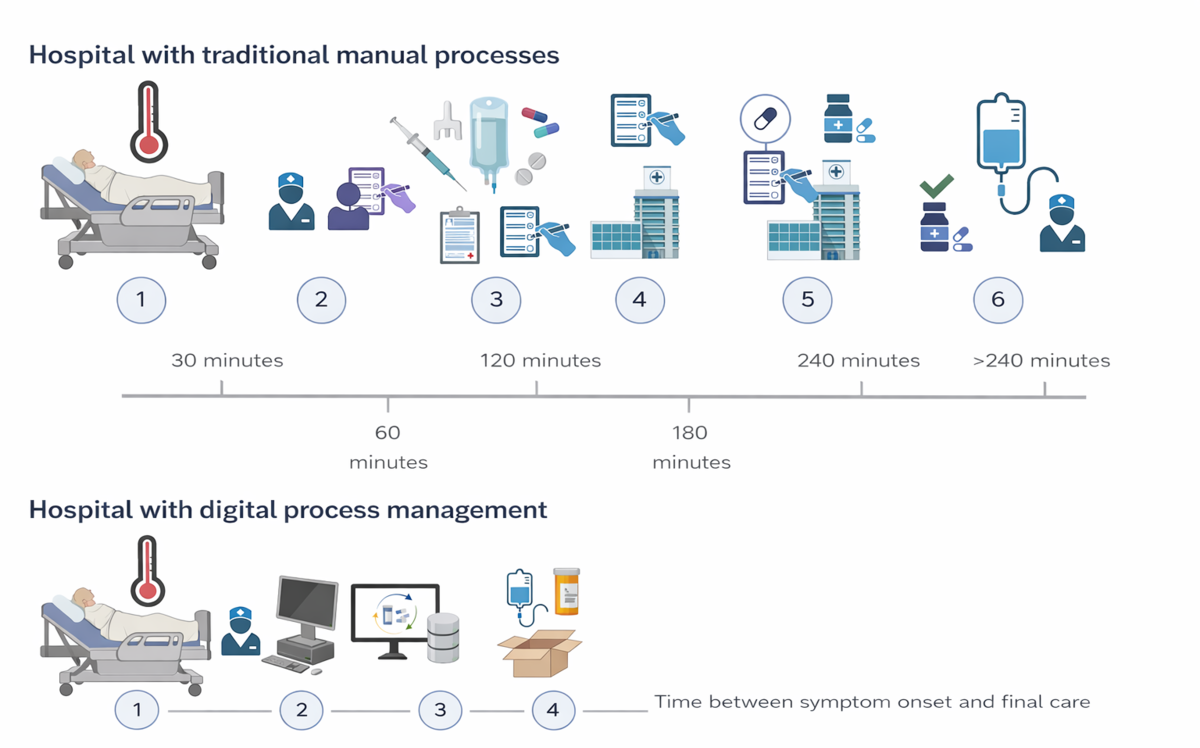
Differences between a hospital that uses traditional physical records (TPRs) and one that use electronic medical records (EMRs) in the duration of antibiotic use and blood-component administration. The sequence of events in the TPR hospital is as follows—step 1: symptom identification; step 2: patient or relatives personally notify health care staff; step 3: symptom registration by nursing or medical personnel; step 4: manual medication request, gathering authorization from supervisors or responsible staff, and verifying medication availability; step 5: approval by senior staff or infectious disease specialists and forwarding the request to the pharmacy; step 6: the pharmacy receives the request, validates prescriptions, dispenses medications, and waits for personnel transport; and step 7: antibiotics administration to the patient. EMR hospital—step 1: symptom identification via automated alerts to nursing staff; step 2: cause identification and digital antibiotic requests with real-time pharmacy validation; and step 3: medication transport via capsule system or designated transport staff, and antibiotics administration to the patient.

The primary outcome was 60-day mortality, defined as death from any cause within 60 days of hospital admission. Secondary outcomes were time from recognition of febrile neutropenia to first antibiotic administration, treatment-related complications during induction, and the number of procedural steps required for urgent antibiotic or blood component delivery. Because our objective was not to assess case-detection capacity but rather to evaluate whether different documentation environments could more efficiently translate clinical decisions into timely pharmacological interventions, workflow analyses focused on execution steps and time stamps after clinical recognition of febrile neutropenia. To characterize workflow differences, we recorded the number of operational steps required for emergent care processes in each hospital. For antibiotic timing analyses, we evaluated a subgroup of 70 patients with complete documentation of febrile episode recognition, antibiotic prescription, and administration time. In the TPR hospital, timing data were obtained from handwritten nursing notes and medication records; in the EMR hospital, they were extracted from electronic time stamps. Figure 1 summarizes the process flow in both institutions. Treatment regimens followed local institutional practice. For ALL, the most frequently used regimens were Hyper-CVAD and CALGB 10403 [16,17]. For AML, the standard 7+3 regimen was the preferred induction approach. For analysis, chemotherapy was categorized as low-, intermediate-, or high-intensity. High-intensity regimens included fludarabine, cytarabine, and granulocyte colony-stimulating factor (FLAG), whereas lower-intensity regimens included azacitidine and low-dose cytarabine [18].

### Statistical Analysis

Because this was a retrospective study that included all consecutive eligible patients treated during the study period, a prospective sample size calculation was not performed. However, considering the observed mortality rates between hospitals, the available sample size provided adequate statistical power to detect clinically meaningful differences between groups. Assuming an early mortality of approximately 30% in the TPR hospital and 10% in the EMR hospital, the sample of 274 patients would provide more than 80% statistical power to detect an absolute difference of at least 20 percentage points between groups at a 2-sided alpha level of .05.

Descriptive statistics were used to summarize baseline characteristics and outcomes. Categorical variables were compared using chi-square or Fisher exact tests, as appropriate, and continuous variables were compared using 2-tailed Student *t* tests or Mann-Whitney *U* tests according to distribution. Missing data were not imputed. Analyses were performed using complete-case information for each variable, and patients without 60-day follow-up were excluded from the primary outcome analysis. Sixty-day survival was analyzed using Kaplan-Meier curves and log-rank tests. To explore factors associated with 60-day mortality in this natural experiment, we fitted a multivariable logistic regression model. Covariates were selected a priori on the basis of clinical relevance, prior literature on early mortality in acute leukemia, baseline imbalances between hospitals, and the need to preserve parsimony relative to the number of deaths observed. The final model included hospital type (EMR vs TPR) as the main exposure of interest, together with age, leukemia type, white blood cell count at diagnosis, prior infection, overweight status, kidney injury, and hepatotoxicity. Odds ratios (ORs) with 95% CIs were reported. A 2-sided *P* value <.05 was considered statistically significant. All analyses were performed using SPSS (version 25.0; IBM Corp).

### Ethical Considerations

This study was conducted in accordance with the Declaration of Helsinki and the institutional procedures of the participating centers. At both hospitals, medical records are routinely reviewed by the institutional medical record committee at discharge as part of standard care and quality control processes. The study used deidentified information derived from these finalized records. The protocol was approved by the Biosafety, Ethics, and Research Committee of the Hospital Regional de Alta Especialidad de Ixtapaluca (protocol HRAEI-CBSI-09-24-2025). Because this was a retrospective analysis of anonymized clinical records, informed consent was waived. All data were handled in a deidentified manner, and no personally identifiable information was accessed or included in the research dataset, in accordance with applicable data protection regulations. No financial or nonfinancial compensation was provided to participants, as this study was based exclusively on retrospective review of existing clinical records.

## Results

### Patient Characteristics

A total of 274 patients were included in the study: 170 (62%) in the TPR group and 104 (38%) in the EMR group. Sex distribution was balanced overall, and no significant difference was observed between hospitals (male sex: 53/104, 51% for EMRs vs 84/170, 49.4% for TPRs; *P*=.45).

The overall mean age was 35 years (range 16-87 years). Patients with AML were older than those with ALL (41 vs 32 years; *P*=.04). ALL was the most frequent diagnosis (183/274, 66.8%), followed by AML (n=46, 16.8%), acute promyelocytic leukemia (n=24, 8.8%), and other myeloid subtypes (n=21, 7.7%).

Regarding comorbidities, 36 (13.1%) patients had diabetes, 18 (6.6%) had hypertension, and 53 (19.3%) were classified as overweight or obese. Secondary leukemia was documented in 9 cases (3.3%), all corresponding to myeloid disease.

Baseline clinical and demographic characteristics by hospital are summarized in Table 1.

**Table 1:** Baseline demographic and clinical characteristics of patients^a^.

			Electronic medical record group (n=104)		Traditional physical records group (n=170)		*P* value	
**Sex, n (%)**								.45
	Male	53 (51)		84 (49.4)				
	Female	51 (49)		86 (50.6)				
Age (years), median (IQR; range)			29.00 (21.00-41.75; 16.00-87.00)		35.00 (17.00-69.00)		.13	
**Age (y), n (%)**								.07
	<35	63 (60.6)		86 (50.6)				
	>35	41 (39.4)		84 (49.4)				
**White blood cell count** **at diagnosis (<30×10^3^** **vs >30×10^3^** **), n (%)**								<.001
	<30×10^3^	79 (76.0)		94 (55.3)				
	>30×10^3^	25 (24.0)		76 (44.7)				
**White blood cell count** **at diagnosis (<100×10^3^** **vs >100×10^3^** **)** **, n (%)**								.005
	<100×10^3^	91 (87.5)		126 (74.1)				
	>100×10^3^	13 (12.5)		44 (25.9)				
**French-American-British** **classification, n (%)**								—^b^
	Acute lymphoblastic leukemia	81 (77.9)		102 (60.0)				
	Acute myeloid leukemia	15 (14.4)		31 (18.2)				
	Acute promyelocytic leukemia	3 (2.9)		21 (12.4)				
	M6 and M7	2 (1.9)		13 (7.6)				
	Biphenotypic	3 (2.9)		3 (1.8)				
**Secondary leukemia, n (%)**								.47
	Present	4 (3.8)		5 (2.9)				
	Absent	100 (96.2)		165 (97.1)				
***BCR::ABL1*** **mutation, n (%)**								.40
	Present	8 (7.7)		16 (9.4)				
	Absent	96 (92.3)		154 (90.6)				
**Central nervous system infiltration** **, n (%)**								.06
	Present	3 (2.9)		14 (8.2)				
	Absent	101 (97.1)		156 (91.8)				
**Diabetes, n (%)**								.25
	Present	16 (15.4)		20 (11.8)				
	Absent	88 (84.6)		150 (88.2)				
**Hypertension, n (%)**								.56
	Present	7 (6.7)		11 (6.5)				
	Absent	97 (93.3)		159 (93.5)				
**Overweight, n (%)**								<.001
	Present	45 (43.3)		8 (4.7)				
	Absent	59 (56.7)		162 (95.3)				
**Previous infection, n (%)**								.002
	Present	41 (39.4)		38 (22.4)				
	Absent	63 (60.6)		132 (77.6)				
**Cytogenetic, n (%)**								—
	Without karyotype	2 (1.9)		36 (21.2)				
	Low risk	48 (46.2)		67 (39.4)				
	Medium risk	06 (5.8)		12 (7.1)				
	High risk	48 (46.2)		55 (32.4)				
Elapsed days between symptoms to diagnosis, n (median; range)			30.00 (17.75-60.00; 0.00-180.00)		30.00 (21.00-60.00; 0.00-360.00)		.17	
Elapsed days between diagnostic to chemotherapy treatment, n (median; range)			6.00 (3.00-9.00: 1.00-29.00)		3.00 (1.00-6.00; 1.00-15.00)		<.001	
**Chemotherapy regimen, n (%)**								—
	Low intensity	1 (1.0)		9 (5.3)				
	Medium intensity	26 (25.0)		128 (75.3)				
	High intensity	77 (74)		33 (19.4)				

^a^The Wilcoxon signed-rank test was used to compare medians of quantitative nonparametric variables for independent groups, and the chi-square test was used for categorical variables. A *P* value of <.05 was considered statistically significant.

^b^Not applicable.

### Baseline Characteristics Before Chemotherapy

Before treatment initiation, among 274 patients, 71 (25.9%) presented with fever and required antibiotic therapy. At admission, the average white blood cell count was 58×10^3^/μL (range 0.3-462×10^3^/μL), without a significant difference between lymphoid and myeloid leukemias (*P*=.35).

Cytogenetic data were available for 159 (58%) patients, with no significant difference between hospitals (*P*=.31). Among 183 patients with ALL, *BCR::ABL1* was detected in 24 (13.1%), and central nervous system infiltration was documented in 17 (9.3%).

Detailed baseline characteristics stratified by hospital are shown in Table 1.

### Treatment Patterns by Hospital

Induction treatment was categorized among 274 patients as low intensity in 10 (3.6%), intermediate intensity in 154 (56.2%), and high intensity in 110 (40.1%). The median time from diagnosis to chemotherapy initiation was 5 (range 1-29; IQR 4) days. High-intensity regimens were more frequently used in the EMR hospital (77/104, 74%) than in the TPR hospital (33/170, 19.4%), whereas intermediate-intensity regimens were more common in the TPR hospital (TPR hospital: 128/170, 75.3% vs EMR hospital, 26/104, 25%). Low-intensity treatment was uncommon in both centers and was mainly reserved for patients who were frail or those with major comorbidities.

Among patients with ALL, most received intermediate-intensity regimens, whereas patients with AML were predominantly treated with standard induction approaches, such as 7+3. Treatment characteristics during induction are summarized in Table 2.

**Table 2:** General characteristics and complications during chemotherapy^a^.

			Electronic medical record group (n=104)		Traditional physical record group (n=170)		*P* value	
**Fever during chemotherapy, n (%)**								.001
	Present	98 (94.2)		136 (80.0)				
	Absent	6 (5.8)		34 (20.0)				
**Neutropenic colitis, n (%)**								<.001
	Confirmed	53 (51.0)		37 (21.8)				
	Absent	51 (49.0)		133 (78.2)				
**Lung pneumonia, n (%)**								.07
	Confirmed	31 (29.8)		35 (20.6)				
	Absent	73 (70.2)		135 (79.4)				
**Systemic candidemia, n (%)**								.23
	Confirmed	16 (15.4)		20 (11.8)				
	Absent	88 (84.6)		150 (88.2)				
**Major hemorrhage, n (%)**								.28
	Presence	27 (26.0)		50 (29.4)				
	Absent	77 (74.0)		120 (70.6)				
**Tumoral lysis syndrome, n (%)**								.56
	Present	13 (12.5)		19 (11.2)				
	Absent	91 (87.5)		151 (88.2)				
**Acute kidney failure, n (%)**								.02
	Present	15 (14.4)		44 (25.9)				
	Absent	89 (85.6)		126 (74.1)				
**Acute liver failure, n (%)**								.03
	Present	19 (18.3)		47 (27.6)				
	Absent	85 (81.7)		123 (72.4)				
**Pancreatitis, n (%)**								.03
	Present	0 (0.0)		7 (4.1)				
	Absent	104 (100.0)		163 (95.9)				
**Toxic megacolon, n (%)**								.11
	Present	1 (0.9)		8 (4.7)				
	Absent	103 (99.1)		162 (95.3)				
Neutropenia (days elapsed), median (range)			23 (18-28)		22 (10.75-30.25)		.42	
Deep neutropenia (days elapsed), median (range)			9 (0-27)		6 (0-37)		.14	
**Positive cultures, n (%)**								<.001
	Present	67 (64.4)		64 (37.6)				
	Absent	37 (35.6)		106 (62.4)				
**Gram-negative bacteria, n (%)**								<.001
	Present	49 (47.1)		40 (23.5)				
	Absent	55 (52.9)		130 (76.5)				
**Gram-positive bacteria, n (%)**								.002
	Present	33 (31.7)		27 (15.9)				
	Absent	71 (68.3)		143 (84.1)				
**Fungal isolation, n (%)**								<.001
	Present	14 (13.5)		06 (3.5)				
	Absent	90 (86.5)		164 (96.5)				
***Clostridium difficile*** **, n (%)**								<.001
	Present	16 (15.4)		5 (2.9)				
	Absent	88 (84.6)		165 (97.1)				
**More than 1 organism in culture, n (%)**								<.001
	Present	31 (29.8)		11 (6.5)				
	Absent	73 (70.2)		159 (93.5)				
**60-day survival** **, n (%)**								<.001
	Live	98 (94.2)		109 (64.1)				
	Death	6 (5.8)		61 (35.9)				

^a^The Wilcoxon signed-rank test was used to compare medians of quantitative nonparametric variables for independent groups, and the chi-square test was used for categorical variables. A *P* value <.05 was considered statistically significant.

### Treatment-Related Complications by Hospital

All patients underwent induction treatment. During this phase, among 274 patients, tumor lysis syndrome occurred in 32 (11.7%), kidney injury in 59 (21.5%), hepatotoxicity in 65 (23.7%), and pancreatitis in 7 (2.6%) patients.

Febrile neutropenia was the most frequent adverse event during induction therapy. It occurred in 98 (94.2%) of 104 patients in the EMR hospital and in 136 (80%) of 170 patients in the TPR hospital (*P*=.001).

Other infectious complications included neutropenic colitis in 90 (32.8%), positive cultures in 131 (47.8%), gram-negative isolation in 89 (32.5%), and gram-positive isolation in 60 (21.9%) patients. Microbiological isolation was reported more often in the EMR hospital.

Major hemorrhage was documented in 77 (28.1%) patients, with no significant difference between hospitals (EMR: 27/104, 26%; TPR: 50/170, 29.4%; *P*=.28).

### Sixty-Day Mortality

Sixty-day mortality for the overall cohort was 24.5% (67/274). Mortality was significantly lower in the EMR hospital (6/104, 5.8%) than in the TPR hospital (61/170, 35.9%; *P*<.001).

Although infectious complications during chemotherapy were more frequently documented in the EMR hospital, severe organ toxicities, such as kidney injury and liver injury, were more common in the TPR hospital.

Mortality was higher among patients with AML (11/46, 23.9%) than among those with ALL (35/183, 19.1%). The leading causes of death were septic shock (41/274, 15%), hemorrhage (20/274, 7.3%), and fulminant liver failure (3/274, 1.1%).

Most deaths from septic shock occurred in patients with ALL, whereas hemorrhagic deaths were concentrated in myeloid leukemias, particularly acute promyelocytic leukemia.

In the multivariable logistic regression analysis, hospital type remained independently associated with 60-day mortality. Care at the EMR hospital was associated with significantly lower odds of death compared with the TPR hospital (OR 0.11, 95% CI 0.05-0.26; *P*<.001). In contrast, kidney injury (OR 1.68, 95% CI 1.29-2.20) and hepatotoxicity (OR 1.49, 95% CI 1.14-1.78) were independently associated with higher odds of 60-day mortality.

### Workflow Measures and Time to Antibiotic Administration

The median duration of neutropenia was 22 (15-30) days overall, and severe neutropenia lasted a median of 7 (3-12) days. Despite a longer duration of neutropenia in the EMR group, only 6 (9%) of the 67 deaths occurred in that hospital.

The TPR hospital required more procedural steps for urgent care processes than the EMR hospital. For antibiotic administration, the TPR hospital required a median of 5 (range 3-7) steps versus 2 steps in the EMR hospital. A similar pattern was observed for blood component requests.

In the subgroup of 70 patients with complete timing documentation, the mean time from recognition of febrile neutropenia to first antibiotic administration was significantly longer in the TPR (272 minutes) hospital than in the EMR hospital (54 minutes; *P*<.001).

### Secondary Survival Analyses and Multivariable Model

**Figure 2:**
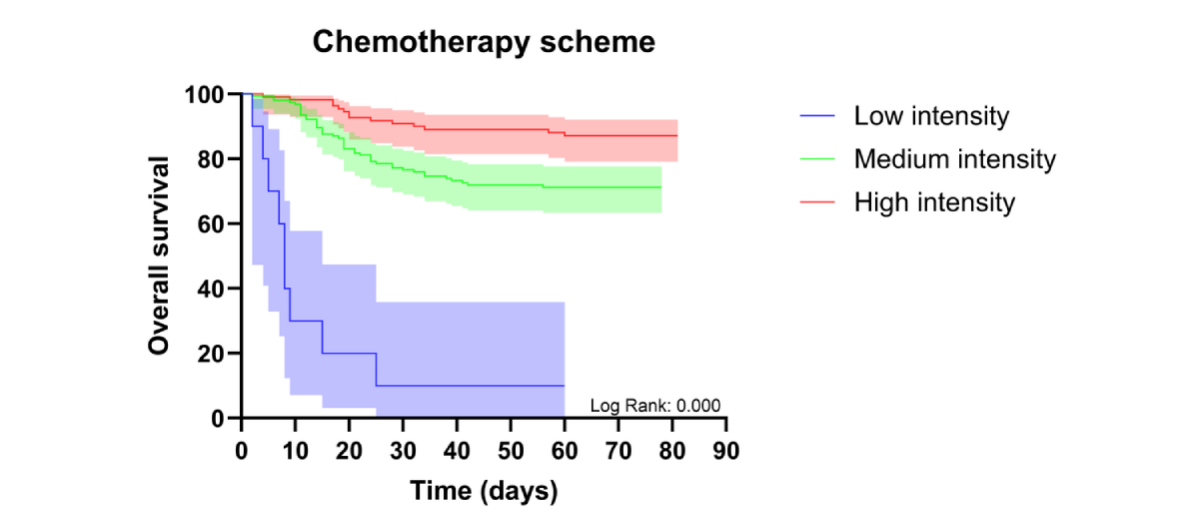
Kaplan-Meier curve comparing 60-day survival according to chemotherapy intensity (secondary analysis).

**Figure 3:**
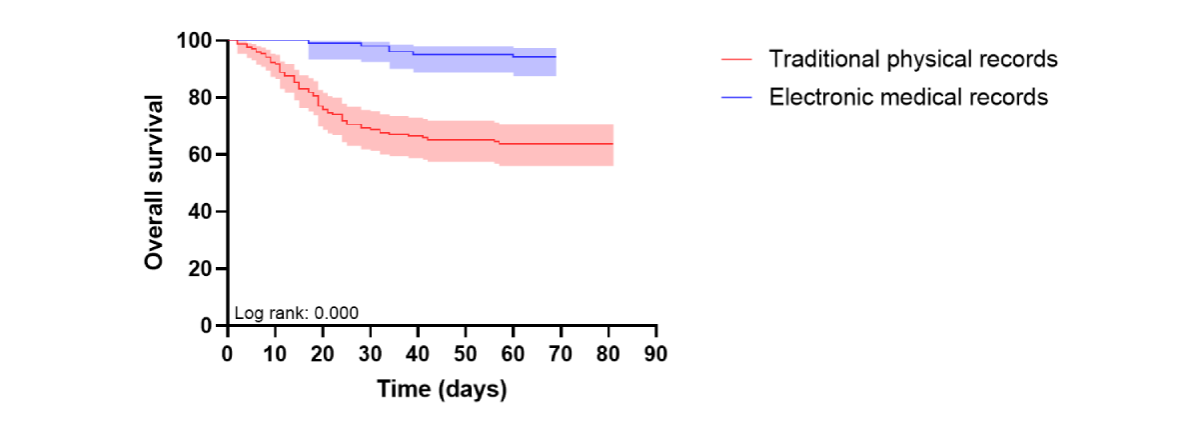
Kaplan-Meier curve comparing 60-day survival between the 2 hospital groups.

Kaplan-Meier analysis of 60-day survival showed poorer survival in patients with AML than in those with ALL. As a secondary analysis, survival also differed according to chemotherapy intensity, which was relevant because treatment intensity varied substantially between hospitals. Survival was also lower in patients treated at the TPR hospital than in those treated at the EMR hospital (Figures 2 and 3).

**Figure 4:**
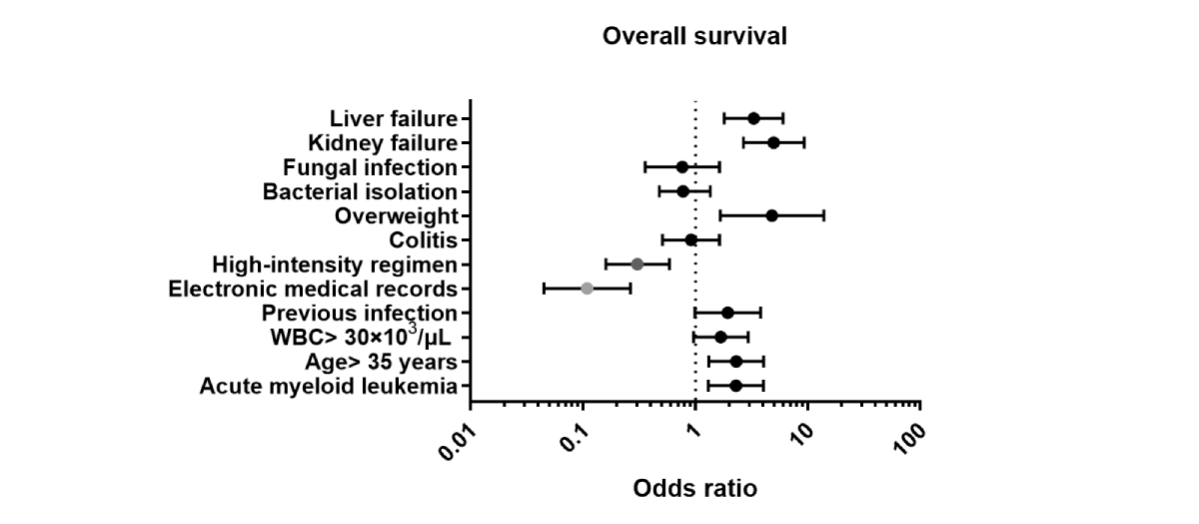
Forest plot of the odds ratios for the main factors associated with 60-day mortality. WBC: white blood cell.

In multivariable logistic regression, hepatotoxicity, kidney injury, overweight status, age older than 35 years, and AML were associated with higher odds of death. By contrast, high-intensity treatment and care at the EMR hospital were associated with lower odds of 60-day mortality. Figure 4 summarizes the ORs for the main variables included in the model.

Overall, these results support the hypothesis that a hospital environment using digitalized workflows may enable faster execution of urgent supportive-care processes and may be associated with lower 60-day mortality; however, the observational design does not allow a causal interpretation.

## Discussion

### Principal Findings

In this retrospective natural experiment, 2 tertiary public hospitals caring for patients with newly diagnosed acute leukemia within the same national setting but with different documentation and medication-management environments were compared. The hospital using an integrated EMR had lower 60-day mortality, shorter times to antibiotic administration in patients with febrile neutropenia, and fewer procedural steps for urgent supportive care than the hospital using TPR. These findings are consistent with the hypothesis that digitalized workflows may improve the timeliness of critical inpatient actions during the first weeks of leukemia treatment, a period in which infectious and hemorrhagic complications strongly influence 60-day mortality.

Several considerations temper the interpretation of these findings. First, this was not an experimental comparison, and the exposure of interest operated at the hospital level rather than the patient level. The 2 hospitals differed in case mix, treatment patterns, and potentially in staffing, clinical experience, referral dynamics, waiting times, bed availability, socioeconomic context, and supportive-care resources. Residual confounding is therefore likely. Second, the study relied on routine clinical and administrative records, which may differ in completeness between paper-based and digital systems. The EMR center may have captured adverse events more completely because of more traceable documentation, whereas some complications may have been underrecorded in paper charts. Third, although all inpatient records are routinely reviewed at discharge by the institutional medical record committee, this quality control process does not eliminate the possibility of information bias in the research dataset. Accordingly, our results should be interpreted as an observed between-hospital association rather than proof of a direct causal effect of the EMR itself.

Despite therapeutic advances, outcomes for acute leukemia in Latin America remain closely tied to health-system performance, including timely diagnosis, rapid treatment initiation, reliable transfusion support, and prompt management of febrile neutropenia [19-24]. In that context, digital tools may matter less as isolated technologies than as enablers of coordination; they can shorten communication chains, document time stamps, reduce transcription delays, and facilitate faster validation of urgent orders.

This interpretation is supported by the marked difference in time to first antibiotic administration observed in our subgroup analysis. For patients with febrile neutropenia, even a modest delay can worsen clinical deterioration, particularly during induction therapy. Therefore, the shorter times observed in the EMR hospital are clinically plausible and may represent one mechanism linking digital workflow to lower 60-day mortality [22-25].

Digital systems may also strengthen accountability and continuous quality improvement. Because records are structured, time-stamped, and easier to audit, EMRs can help identify operational bottlenecks that remain largely invisible in paper-based systems. In hematology-oncology programs, where care depends on repeated coordination among physicians, nurses, pharmacists, blood bank staff, and laboratory services, this additional traceability may be especially valuable [26-29].

At the same time, the use of an integrated EMR should not be viewed as the only explanation for the observed differences. Digitalization likely functions as one component of a broader institutional environment that includes organizational culture, training, responsiveness of ancillary services, and medication availability. Therefore, the lower mortality observed in the EMR hospital may reflect both the digital platform itself and the systems-level practices that accompany its use.

The broader regional literature supports this interpretation. Studies from Latin America consistently show that standardized pathways, timely supportive care, and better-organized referral and treatment networks can improve survival in hematologic malignancies [30-32]. Our results add to that literature by suggesting that hospital digitalization may be one practical strategy for strengthening these pathways in public-sector settings, but they do not establish that digitalization alone explains the observed mortality difference.

Rather than framing EMRs only as documentation tools, our findings support evaluating them as part of institutional redesign aimed at reducing preventable deaths within 60 days of admission. In acute leukemia, the clinically relevant question is not only whether data are stored electronically but also whether digital systems shorten the interval between clinical recognition, order entry, validation, and treatment delivery.

From a policy perspective, this distinction is important. Public hospitals operating with limited resources may derive meaningful benefit from digital tools when digitalization is linked to workflow simplification, clinical accountability, and rapid-response processes. Therefore, investments in EMRs are likely to be most effective when accompanied by staff training, supportive-care protocols, antimicrobial stewardship, and transfusion logistics optimization.

A major strength of this study is that it compares 2 real-world tertiary public hospitals caring for patients with acute leukemia within the same national health context but with clearly different documentation and medication-management models. The analysis also incorporated clinically relevant covariates associated with 60-day mortality and examined not only outcome differences but also a plausible process-of-care mechanism, namely time to antibiotic administration.

This study also has important limitations. Its retrospective design introduces the possibility of selection bias and information bias. Because only 2 hospitals were included, the analysis is best interpreted as a natural experiment and has limited generalizability. Residual confounding is likely, because unmeasured differences between centers—including staffing, referral complexity, waiting times, socioeconomic context, supportive-care capacity, and other institutional characteristics—may have influenced mortality. Although medical records at discharge are routinely reviewed by the institutional medical record committee, documentation quality may still differ between paper and digital systems. No formal duplicate abstraction or interrater reliability assessment was performed for the research dataset. Missing data were handled through complete-case analysis rather than imputation, and the antibiotic timing analysis was restricted to a subgroup with complete documentation, which may have introduced additional selection bias.

### Future Directions

Future research should validate these findings in prospective multicenter cohorts, incorporate standardized abstraction procedures across institutions, and include more detailed measures of workflow, staffing, supportive-care capacity, and case severity. Studies evaluating cause-specific mortality, time to transfusion support, and cost-effectiveness would also help clarify which components of digital implementation have the greatest clinical impact.

Further work should also explore how digital systems interact with staff training, antimicrobial stewardship, transfusion logistics, and clinical decision support in patients with hematologic malignancies. Such studies would help distinguish the specific contribution of the EMR platform from the contribution of the broader institutional practices that accompany digital care.

### Conclusions

In this retrospective natural experiment, the hospital using an integrated EMR had a lower 60-day mortality and faster antibiotic administration than the hospital using TPR in patients with acute leukemia. These results support the hypothesis that digitalized workflows may improve the timeliness of urgent supportive care during induction therapy.

However, the findings should be interpreted with caution because the study was observational and compared 2 nonequivalent institutions using routine clinical records. Prospective multicenter studies are needed to confirm whether the observed between-hospital differences persist after more detailed adjustment for case mix, workflow, and institutional characteristics, and to identify which components of digital care are most relevant for improving outcomes.

## Abbreviations

ALL: acute lymphoblastic leukemia
AML: acute myeloid leukemia
EMR: electronic medical record
FLAG: fludarabine, cytarabine, granulocyte colony-stimulating factor
HITECH: Health Information Technology for Economic and Clinical Health
OR: odds ratio
TPR: traditional physical record

## Data Availability

Data are available from the corresponding author on reasonable request, subject to institutional privacy and confidentiality restrictions.
